# Dietary interventions in acute kidney injury: From molecular mechanism to clinical trials

**DOI:** 10.1113/EP092817

**Published:** 2026-03-25

**Authors:** Felix C. Koehler, Pedro Rojas‐Morales, Roman‐Ulrich Müller

**Affiliations:** ^1^ Department II of Internal Medicine and Center for Molecular Medicine Cologne, Faculty of Medicine and University Hospital Cologne University of Cologne Cologne Germany; ^2^ Cologne Excellence Cluster on Cellular Stress Responses in Aging‐Associated Diseases (CECAD), Faculty of Medicine and University Hospital Cologne University of Cologne Cologne Germany; ^3^ Department of Internal Medicine (Nephrology) & Einthoven Laboratory of Vascular and Regenerative Medicine Leiden University Medical Center Leiden Netherlands; ^4^ Center for Rare Diseases Cologne, Faculty of Medicine and University Hospital Cologne University of Cologne Cologne Germany; ^5^ Department of Nephrology, Medical Faculty University Hospital Düsseldorf, Heinrich‐Heine‐University Düsseldorf Düsseldorf Germany

**Keywords:** acute kidney injury, dietary interventions, resilience

## Abstract

Ageing impairs renal resilience with an elevated risk of frequent and harmful acute kidney injury (AKI) that causes substantial morbidity and mortality among hospitalized patients. Since different damaging stimuli at the molecular, cellular and functional level contribute to this loss in kidney function, AKI's pathophysiology is heterogeneous and complex, and consequently, the development of pharmacological approaches is lagging behind. On the other hand, dietary interventions, such as caloric restriction, periodic fasting, ketogenic diets and restriction of sulfur‐containing amino acids have shown immense potential in preserving kidney function in various rodent models of AKI. Deciphering the underlying, but conserved, molecular mechanisms or renal resilience in mice and humans will help to pave the way to successful transfer to the patient setting and may allow the identification of druggable targets. As these diet‐induced protective effects of preconditioning are not limited to the kidney, dietary interventions may even be extended to other tissues like the brain or the heart in the context of AKI and beyond.

## Abstract (highlights)

- **What is the topic of this review?**
  Dietary interventions show immense potential in preventing kidney function decline from various damaging stimuli in rodent models. However, translation to kidney disease patients is still lagging behind, calling for the study of underlying and conserved molecular mechanisms in mice and humans.
- **What advances does it highlight?**
  This narrative review presents a comprehensive overview of current molecular mechanistic concepts of diet‐induced resilience in animal models and their recapitulation in humans, whilst reflecting the heterogeneous pathophysiology in acute kidney injury.

## 1 ACUTE KIDNEY INJURY – A LIFE‐THREATENING AGEING‐ASSOCIATED DISORDER

One of the key organs affected by ageing is the kidney. A pivotal challenge in ageing‐associated kidney disease is the elevated risk of acute kidney injury (AKI), which is in turn one of the most common complications among hospitalized patients. Being an ageing‐associated disorder, its incidence will increase even further in the future due to demographic change.

Importantly, AKI is associated with substantial mortality of approximately 15%, even in a non‐intensive care setting (Ostermann et al., 2025). Non‐recovery of kidney function and progression to chronic kidney disease (CKD) after AKI are common. CKD itself currently affects close to 100 million Europeans and is projected to become the fifth leading cause of death worldwide by 2040 (Vanholder et al., 2021).

## 2 KIDNEY HETEROGENIC PATHOPHYSIOLOGY

**FIGURE 1:**
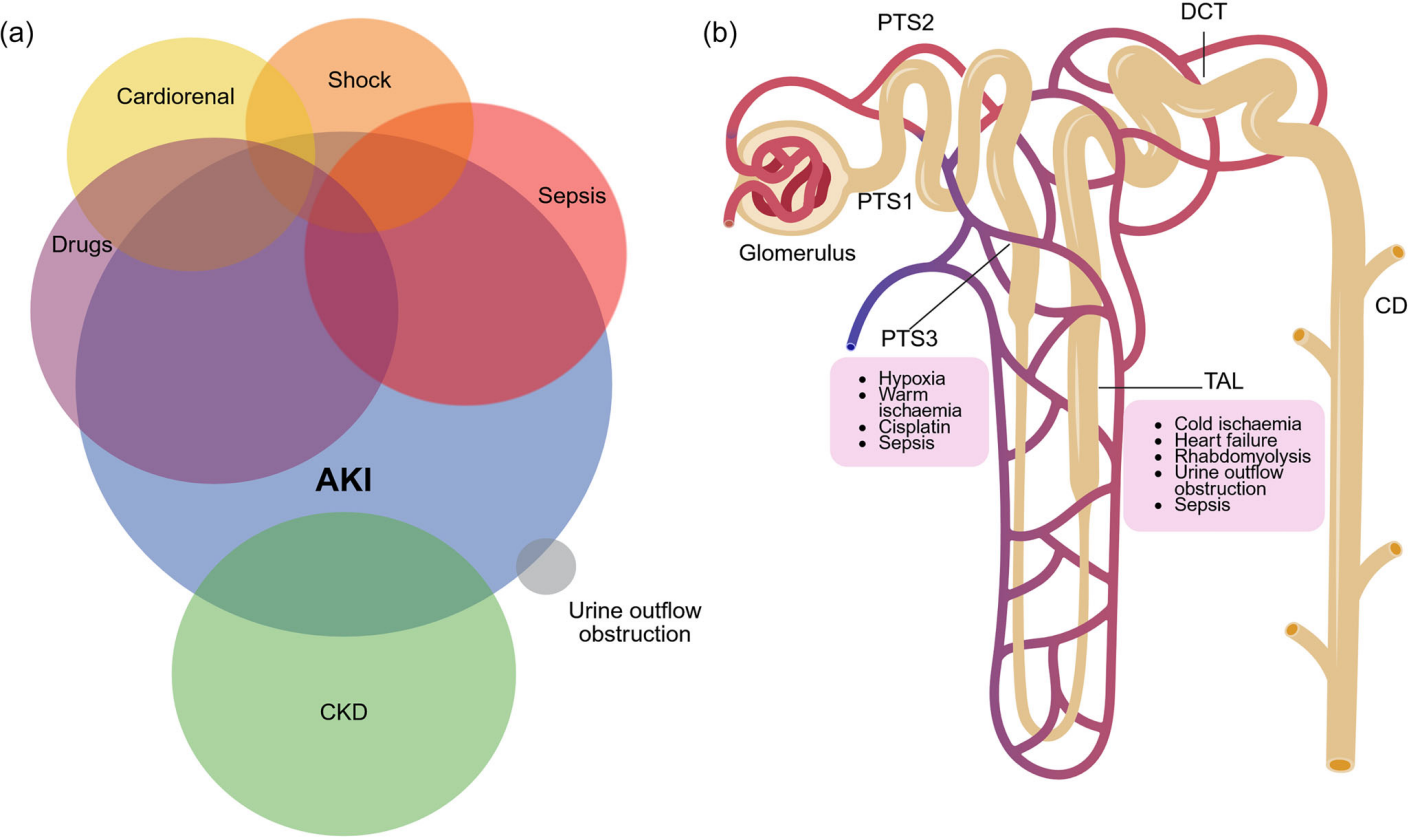
The diverse spectrum of acute kidney injury (AKI) in the clinic is reflected by its heterogenous pathophysiology at the molecular, cellular and functional levels in the kidney. (a) Frequently occurring, selected, key aetiologies of AKI may overlap in a combined causality. Size of circles reflects the frequency of each AKI syndrome (Ronco et al., 2019). (b) Susceptibility of nephron segments to the different, selected, key aetiologies for AKI. The S_3_ segment of proximal tubules (PTS3) is particularly vulnerable to hypoxia and warm ischaemia. In contrast, the thick ascending limb (TAL) is more susceptible to cold ischaemia, heart failure rhabdomyolysis and urine outflow obstruction. AKI, acute kidney injury; CD, collecting duct; CKD, chronic kidney disease; DCT, distal convoluted tubules; PTS1, proximal tubules segment S_1_; PTS2, proximal tubules segment S_2_; PTS3, proximal tubules segment S_3_; TAL, thick ascending limb. Figure created with BioRender.com.

AKI is defined and diagnosed in the clinic based on an increase in serum creatinine or a decrease in urinary output (Khwaja, 2012). Thus, AKI can be considered a clinical syndrome rather than a distinct disease, and the main goal in clinical evaluation is to identify and address the underlying aetiology (Ostermann et al., 2025; Scholz et al., 2021). The traditional simplistic taxonomy classifying the aetiology of AKI according to semi‐anatomical categories (pre‐renal, intrinsic, and post‐renal AKI) is now giving way to more specific syndromic descriptions such as cardiorenal, hepatorenal, nephrotoxic, ischaemic and sepsis‐associated AKI among other entities (Ostermann et al., 2025). This increased specificity reflects that these syndromes have their own unique pathophysiology and treatment approaches. However, these common AKI syndromes often coexist in the clinic (Figure 1a) (Mehta et al., 2016).

As the pathophysiology of AKI is heterogeneous and complex, divergent responses (simultaneously or in sequence) at the molecular, cellular and functional levels have been discovered in preclinical studies. To begin with, the various nephron segments differ in their blood, metabolic and oxygen supplies as well as energy demands, which accounts for variable regional susceptibilities of the kidney tubular cells to different damaging stimuli. In this context, the ability to activate mechanisms of cellular stress‐resistance and/or initiate repair programmes varies among the different tubular cell types and is another reason for the heterogenic pathophysiology (Scholz et al., 2021).

## 3 VULNERABILITY OF NEPHRON SEGMENTS AND DIFFERENT MODES OF KIDNEY INJURY

The metabolic rate of the kidneys is as high as that of the heart in healthy humans, thus reflecting its enormous energy demands, since more than 70% of the glomerular filtrate is reabsorbed in proximal tubules (Wang et al., 2010). This is reflected by the high mitochondrial density and oxygen consumption rate in the kidneys (Friederich‐Persson et al., 2018). The high energy demands of tubular epithelial cells in the kidney is met by metabolizing a wide range of substrates with only a small capacity for glycolysis (Scholz et al., 2021). Especially, the S_3_ segment of proximal tubules (PTS3) is at a high risk for energy shortage in the context of hypoxia due to its localization in the outer medulla paired with its low anaerobic glycolytic capacity. Thus, PTS3 is the major site of injury in hypoxia and warm kidney ischaemia (Heyman et al., 2020). Another site of injury in AKI is the thick ascending limb (TAL), which is particularly sensitive to cold ischaemia. Furthermore, the TAL is more susceptible to salt depletion, heart failure, rhabdomyolysis and urine outflow obstruction as compared to the PTS3 (Scholz et al., 2021). With regard to frequent septic AKI, its pathognomonic cellular injury patterns are less clear, as its pathophysiology is overlapping and complex, since it combines inflammatory reactions and haemodynamic changes in addition to metabolic reprogramming (Figure 1B) (Lelubre & Vincent, 2018).

## 4 SYSTEMIC CONSEQUENCES OF AKI: ORGAN CROSSTALK AND IMMUNE DYSREGULATION

**FIGURE 2:**
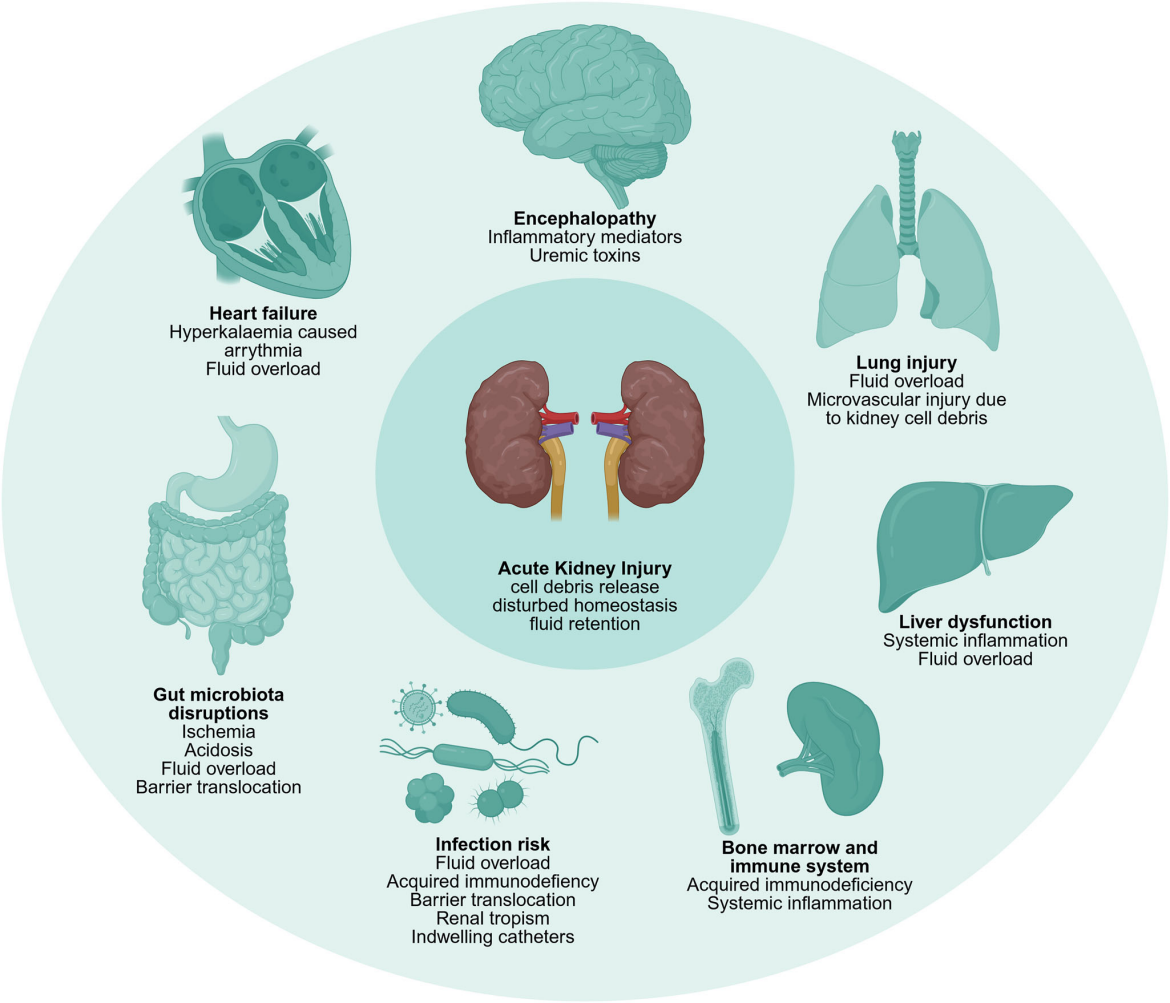
Systemic consequences caused by AKI: organ crosstalk and immune dysregulation. AKI‐induced distant organ effects are not limited to decline in organ function, but also include microvascular inflammation and coagulation, cell apoptosis, oxidative stress and transcriptional responses. Figure created with BioRender.com.

As overall body homeostasis is closely connected to kidney health, kidney failure affects most organ systems of the body (Figure 2). For example, AKI heavily disturbs the body's fluid and electrolyte homeostasis triggered by the decline of the glomerular filtration rate (GFR) paired with the parallel activation of the renin–angiotensin system.

Many of the AKI‐related uraemic toxins derive from gut microbiota, such *p*‐cresyl sulfate and indoxyl sulfate, and cause harm to the body (Vanholder et al., 2014). On the other hand, AKI and its sequelae such as acidosis, azotaemia or intestinal ischaemia substantially change the composition of the faecal microbiome (Gharaie et al., 2023). This alters the microbiotal secretome, a fact which is of interest as microbially generated metabolites are needed for human health (Koehler et al., 2024; Meijers et al., 2019).

However, AKI not only changes the intestinal flora but also harms intestinal barrier integrity and immunological fitness (Andersen et al., 2017; Meijers et al., 2019). As AKI additionally affects the immune system, ultimately causing an acquired immunodeficiency paired with systemic inflammation, this increases the risk of infectious complications in patients suffering from AKI (Figure 2). Of note, the risk for severe infectious diseases, eventually resulting in sepsis, remains high up to 2 weeks after AKI due to ongoing immunosuppression, although the kidney function may already have fully recovered. As sepsis is the most common cause of AKI, a vicious circle may arise, where sepsis‐induced immunodeficiency is prolonged by sepsis‐induced AKI and vice versa (Kellum et al., 2012).

Besides disturbed homeostasis, renal cell death further results in cell debris release that causes not only systemic inflammation, but also microvascular injury in different organs, such as brain, heart, liver and the lung, as summarized in Figure 2.

## 5 DIET‐INDUCED ORGANISMAL RESILIENCE – A KEY STRATEGY FOR KIDNEY PROTECTION?

**FIGURE 3:**
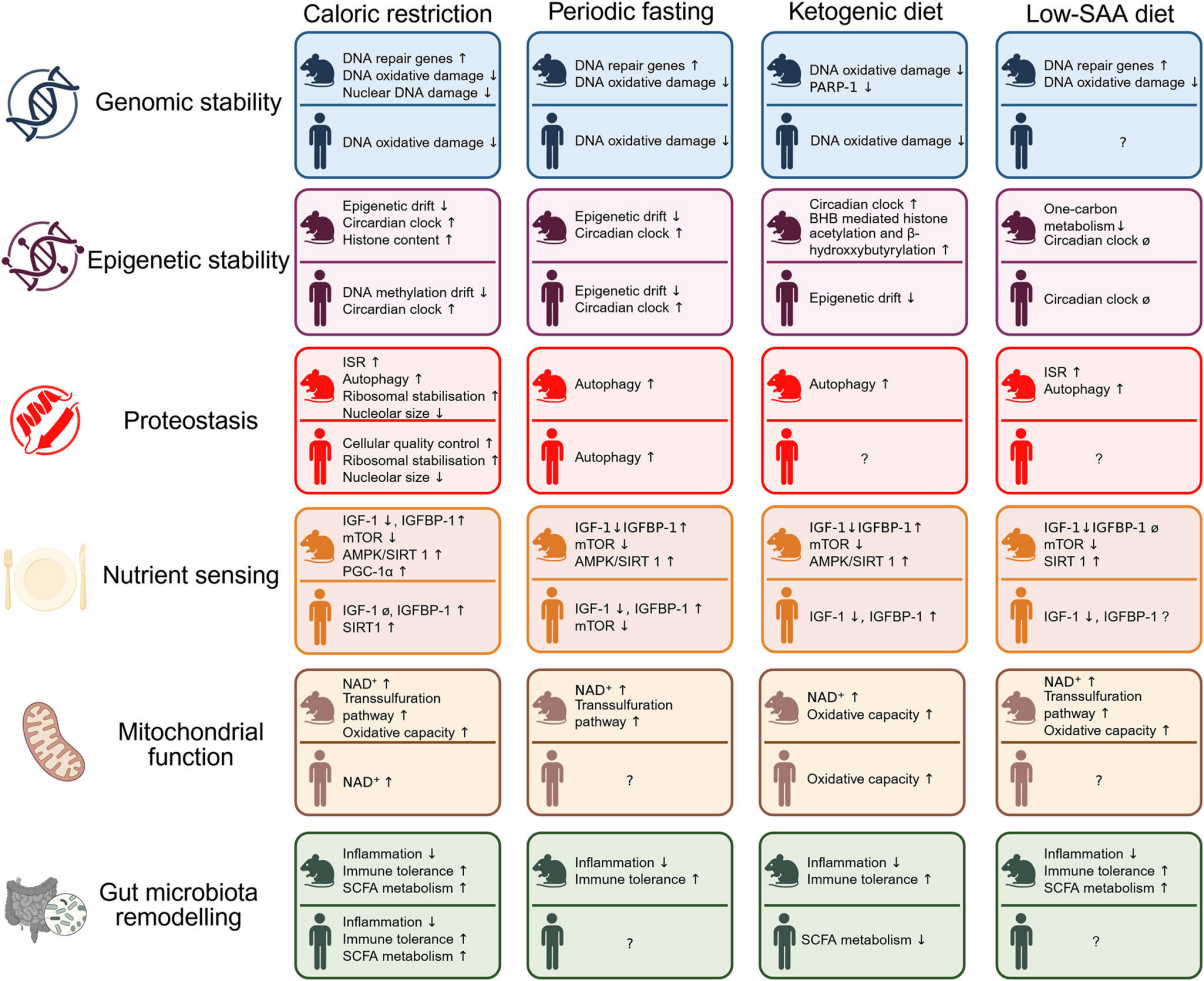
Overview of diet‐induced effects on hallmarks of cellular stress‐resistance observed in rodent models in addition to humans adhering to specific dietary pattern in clinical trials. A summary of the refence literature is available as Supporting Information. AMPK, AMP‐activated protein kinase; BHB, β‐hydroxybutyrate; IGF‐1, insulin growth factor 1; IGFBP‐1, insulin growth factor binding protein 1; ISR, integrated stress response pathway; mTOR, mechanistic target of rapamycin; PGC‐1α, peroxisome proliferator‐activated receptor γ coactivator 1‐α, SCFA, short chain fatty acids; SIRT1, sirtuin 1. Figure created with BioRender.com.

Despite this considerable burden, effective therapeutic approaches, including prevention strategies, are lacking (Ostermann et al., 2025). This calls for new concepts to fight an old threat. Specific metabolic changes ameliorated by different dietary interventions have recently been identified as being linked to an extended life‐ and health‐span mediating by an increase in both organismal and cellular resilience. As impaired cellular stress‐resistance is a key driving factor for AKI, these dietary interventions may add substantially to our therapeutic armamentarium to fight both AKI and its systemic consequences. Protective dietary strategies activate evolutionarily conserved metabolic pathways in animal models and humans, an important pre‐requisite for potential translation to the patient setting and will be further reviewed below (Figure 3) (Koehler et al., 2021, 2024).

## 6 CALORIC RESTRICTION

Caloric restriction (CR) – usually defined as a reduction in caloric intake by 10–40% – is the simplest dietary protocol. However, CR shows an unexpectedly powerful potential to protect from kidney damage in various rodent models of ischaemia and toxicity‐caused AKI (Johnsen et al., 2020; Koehler et al., 2022; Mitchell et al., 2010; Späth et al., 2023). Whether CR rescues mice from sepsis‐caused AKI still remains elusive. Nonetheless, short‐term CR improves the survival outcome in polymicrobial sepsis and sterile endotoxaemia in rodent models (Starr et al., 2016).

Current concepts of CR‐mediated cellular stress‐resistance include molecular, cellular and systemic mechanisms as the basis of organ protection (Koehler et al., 2021). To begin with, CR directly affects genome stability mediated by a rise in DNA repair genes and a reduction of oxidative as well nuclear DNA damage (Cabelof et al., 2003). Of note, a reduction in oxidative DNA damage could be recapitulated in humans adhering to limited caloric intake over 6 months (Heilbronn et al., 2006). Genomic stability is further accompanied by epigenetic stability mediating cellular resilience, as CR affects the circadian clock and the DNA methylation drift, even in humans (Waziry et al., 2023).

The activation of tryptophan metabolism and the production of NAD is another important molecular concept of CR‐mediated stress‐resistance in AKI (Späth et al., 2023). In this context, the important family of NAD‐dependent deacetylases modifies protein deacetylation of key metabolic enzymes (Ralto et al., 2020). The acetylation status is considered to orchestrate metabolic pathway activity ultimately resulting in resilience (Späth et al., 2023). A well‐known example is the NAD‐dependent type III deacetylase sirtuin 1 (SIRT1), which is linked to organ protection from ischaemia in the kidney and beyond (Fan et al., 2013; Späth et al., 2023).

Moreover, specific nutrient‐sensing pathways are considered to be another key orchestrator in diet‐induced kidney protection (Koehler et al., 2021; Scholz et al., 2021). Thus, mechanistic target of rapamycin (mTOR) and AMP‐activated protein kinase (AMPK) are considered the two most studied nutrient‐sensing pathways mediating kidney protection, as they regulate cellular metabolism and homeostasis, including mitochondrial health (González et al., 2020; Jiang et al., 2012). As mitoptosis, mitophagy and mitochondrial permeability transition driven necrosis occur in AKI, mitochondrial health is considered another key orchestrator of the CR‐induced organ protection in mice and humans (Dugbartey, 2024; Koehler et al., 2024). Strikingly, AMPK increases NAD levels as another mechanism of kidney protection highlighted above (Cantó et al., 2009).

Comparative bulk RNAseq has additionally identified Cyp4a12a, a cytochrome that is strongly downregulated in response to CR in male mice (Hoyer‐Allo et al., 2022). In line with this, CR significantly reduced the tissue concentration of its product, 20‐hydroxyeicosatetraenoic acid (20‐HETE), and supplementation of 20‐HETE, in turn, diminished the CR‐mediated kidney protection. As Cyp4a12a is exclusively expressed in male mice, this may be an explanation for the different sex‐specific pattern of AKI observed in rodent models (Hoyer‐Allo et al., 2022). Beyond these molecular concepts of CR‐induced resilience, the nucleolus is considered a key cellular player in CR‐mediated kidney protection. The nucleolus is a central stress‐sensor and its integrity is essential for life and health‐span extension; small nucleoli are a cellular hallmark of longevity conserved across taxa (Tiku et al., 2017). Since all organisms respond to cellular stress by downregulating the synthesis of rRNA and ribosomal biogenesis, the nucleolus is a central hub in coordinating the cellular stress‐response in the kidney and beyond. As rRNA synthesis is a very energy consuming cellular process, switching off transcription of rRNA genes is an effective and conserved strategy to save energy and coping with acute cellular damage to maintain cellular integrity and homeostasis as well as protein and RNA quality control (Grummt, 2013).

In rodent studies, CR is typically implemented by providing food once daily at a fixed time during the day, followed by extended fasting periods (Hoyer‐Allo et al., 2022; Koehler et al., 2022; Mitchell et al., 2010; Späth et al., 2023). Consequently, CR regimens in rodents not only reduce overall caloric intake but also incorporate elements of time‐restricted feeding or intermittent fasting (Koppold et al., 2024; Longo et al., 2021). In humans, by contrast, calorie intake may be solely reduced without adding elements of time‐restricted feeding as discussed further below (Grundmann et al., 2020, 2018).

## 7 PERIODIC FASTING

Periodic fasting is characterized as a dietary restriction period – either a complete restriction of food (water only) or a severe restriction of caloric intake for a prolonged time (usually more than 2 days) – that is followed by an unrestricted ad libitum refeeding period (Longo et al., 2021). Several studies have shown that various fasting protocols – both chronic and intermittent – enhance resilience against AKI in rodents  (Kim et al., 2024; Koehler et al., 2022; Mitchell et al., 2010; Rojas‐Morales et al., 2019). These protective effects are primarily mediated by increased antioxidant defences, reduced inflammation and improved mitochondrial function, ultimately helping to prevent progression to CKD (Rojas‐Morales et al., 2020).

Fasting‐mimicking diets (FMD) are the most common periodic fasting regimens designed to cause fasting‐like beneficial effects, while providing sufficient macro‐ and micronutrient intake to limit the burdens of water‐only fasting (Brandhorst et al., 2015). FMD achieves its well‐known beneficial effects through its impacts on insulin signalling, including the reduction of insulin‐growth factor 1 (IGF‐1), whilst increasing insulin‐growth factor binding protein 1 (IGFBP‐1) in mice (Brandhorst et al., 2015). More importantly, in contrast to CR, FMD is reported to reduce levels of IGF‐1 even in humans (Fontana et al., 2016; Wei et al., 2017). In turn, insulin/IGF‐1 signalling is known to mediate diet‐induced kidney protection, and FMD efficiently protects kidney function from renal ischaemia–reperfusion injury (IRI) in rodents (Koehler et al., 2022, 2021; Mitchell et al., 2010). As CR may lead to malnourishment, limiting its widespread use in multimorbid and frail nephrology patients, FMD is considered a promising alternative beyond CR that has already been examined successfully in clinical trials beyond nephrology (Longo et al., 2021).

## 8 KETOGENIC DIETS AND THERAPEUTIC KETOSIS

As CR and FMD results in ketosis in rodents and considering the recently shown beneficial effects of ketogenic diets (KD) in kidney disease, KDs are considered another promising dietary strategy beyond CR (Cukoski et al., 2023; Koehler et al., 2022; Rojas‐Morales et al., 2022). However, the protective effects induced by preconditioning with KDs appear to be less profound in comparison to the other reviewed dietary preconditioning regimens as established in parallel testing using a mouse model of renal IRI (Koehler et al., 2022). Nonetheless, KDs results in improved survival and preserved kidney function when compared to ad libitum‐fed control mice. KD‐induced tolerance to acute renal ischaemic damage is reported to be stronger in rats (Koehler et al., 2022; Rojas‐Morales et al., 2022). These observed differences in nephroprotection call for further investigation, in particular, as KDs recently showed beneficial effects in critically ill patients with sepsis, the most common cause of AKI in hospitalized patients (Figure 1a) (Ostermann et al., 2025; Rahmel et al., 2024).

Improved immune tolerance accompanied with the reduction of inflammation is a possible explanation for the observed benefits of KDs in rodents and, in particular, in septic patients (Rahmel et al., 2024; Rojas‐Morales et al., 2022). In this context, the diet‐induced impact on gut microbiota has recently been recognized as a central modulator of cardiovascular and renal disease (Gharaie et al., 2020; Meijers et al., 2019; Ross et al., 2024). Of note, KDs deplete bifidobacteria and inhibit their growth through ketone bodies. This leads to a reduction in pro‐inflammatory TH17 cells in mice and humans and, in turn, indicates the close interaction between gut microbiota and the immune system in response to dietary interventions (Ang et al., 2020). Furthermore, the bacterial key biosynthethetic enzyme of lipid A, a critical lipopolysaccharide (LPS), is reduced in response to dietary interventions. LPSs are well‐known cell wall components of gram‐negative bacteria that promote inflammation and exacerbate, in particular, sepsis‐induced AKI (Fabbiano et al., 2018). In turn, reduced levels of LPSs are linked to immune tolerance (Koehler et al., 2021).

## 9 SULFUR‐CONTAINING AMINO ACID RESTRICTION

Besides tryptophan metabolism, reduced levels of sulfur‐containing amino acids (SAA) are considered an orchestrator of the CR‐induced benefits. In line with this, these benefits are lost when mice are supplemented with SAAs while restricting the overall caloric intake (Hine et al., 2015). As dietary restriction of the SAAs methionine and cysteine is feasible without malnourishment, low‐SAA diets are considered a promising novel tailored dietary interventions beyond CR in the clinic (Koehler et al., 2021, 2024; Osterholt et al., 2022). In line with these findings, dietary restriction of SAAs was found to be as protective as CR in a comparative study using a mouse model of renal IRI (Koehler et al., 2022).

Of note, cysteine is a non‐essential SAA and can be made from homocysteine via the so‐called transsulfuration pathway (TSP). In the last years, cysteine metabolism and the TSP have been identified as a central mechanism of diet‐induced resilience in various model organisms. Especially, endogenous generation of cytoprotective hydrogen sulfide (H_2_S) by specific diet‐induced alterations in cysteine metabolism is considered a conserved mechanism to improve organismal stress resistance (Hine et al., 2015; Koehler et al., 2022). H_2_S augments anti‐inflammatory mechanisms, reduces reactive oxygen species and changes lipid metabolism (Kohl et al., 2019). In addition, H_2_S modulates the structure and function of many proteins through protein persulfidation (S‐sulfhydration), an evolutionarily conserved post‐translational modification of cysteine residues that is linked to organismal resilience (Bithi et al., 2021; Hine et al., 2015). The primary sources of H_2_S generation, in turn, are various metabolites that arise from cysteine metabolism (Koehler et al., 2022; Kohl et al., 2019).

In addition to the H_2_S‐producing branch, there are two further pathways involved in cysteine metabolism: (1) oxidative cysteine breakdown and (2) γ‐glutamylcysteinylglycine (also called glutathione, GSH) biosynthesis (Koehler et al., 2022). Whereas the flux from cysteine to taurine is decreased, a parallel rise in oxidative cysteine catabolism in favour of GSH has been observed in diet‐induced kidney protection from ischaemic damage (Koehler et al., 2022). This links cysteine metabolism to ferroptosis, an iron‐dependent cell death process due to lipid peroxide accumulation present in AKI (Abu‐Remaileh et al., 2017; Tonnus et al., 2025).

## 10 MICRONUTRIENTS

The World Health Organization (WHO) defines micronutrients as vitamins and minerals required by the body in very small amounts that are nonetheless essential for maintaining health. In the context of kidney health and disease, several minerals – including zinc, iron, copper and selenium – as well as vitamins such as vitamin A, the vitamin B complex (including vitamins B6, B12 and folic acid), vitamin C and vitamin D, in addition to amino acids, play important roles. Micronutrient status is markedly disrupted in patients with acute kidney injury (AKI), particularly in those requiring renal replacement therapy (RRT), compared with healthy individuals (Cameron et al., 2024; Ostermann et al., 2020).

Mechanistically, micronutrients with antioxidant properties ameliorate ischaemic kidney damage by scavenging reactive oxygen species (Mauerhofer et al., 2021). For example, zinc chloride (ZnCl_2_) is reported to protect kidney function against the effects of reactive oxygen species by enhancing the activity of antioxidant enzymes and increasing GSH levels (Hadj Abdallah et al., 2018). Similarly, selenium and its intermediate hydrogen selenide exert direct antiferroptotic effects, rendering cells resistant to oxidative stress. The conversion of selenite to hydrogen selenide via the selenocysteine biosynthetic pathway highlights a close link between selenium and cysteine metabolism (Lee et al., 2024).

Vitamin A (retinol) and its metabolite retinoic acid have been reported to influence cell repair of proximal tubules after damaging stimuli in rodents (DiKun & Gudas, 2023). High doses of vitamin B12 (cyanocobalamin) protect kidneys against IRI, as it scavenges reactive oxygen species and decreases renal superoxide formation in response to ischaemia (Li et al., 2020). In line with this, pretreatment with folic acid, a member of the vitamin B complex, limited tissue damage and preserved kidney function in rodents (Hamed et al., 2024). Regarding vitamin C, beneficial effects were observed when it was administered during reperfusion in rats undergoing renal IRI, primarily by attenuating oxidative stress and inflammation, whereas pretreatment with vitamin C conferred only mild protection (Ko et al., 2024).

Clinical data are available in the context of vitamin B1 (thiamine) treatment in septic patients. Vitamin B1 plays a pivotal role in mitochondrial respiration and overall mitochondrial fitness, processes that have been associated with kidney protection (Koehler et al., 2021). However, current randomized controlled trials have reported mixed results concerning kidney protection in septic intensive care unit (ICU) patients. Nonetheless, the ICU stay was shortened in vitamin B1 treated patients, and no adverse effects were reported, indicating that thiamine supplementation is safe (Moskowitz et al., 2017, 2023; Woolum et al., 2018).

## 11 GUT MICROBIOTA AS MEDIATORS OF DIET‐INDUCED RENAL RESILIENCE

Gut microbiota are increasingly known for their pivotal role in kidney health and disease (Andrade‐Oliveira et al., 2015; Gharaie et al., 2023). Dietary interventions, in turn, regulate the diversity, composition as well as function of gut microbiota (Ross et al., 2024). Anaerobic gut microbiota produce various beneficial metabolites, such as short‐chain‐fatty acids (SCFA), through saccharolytic fermentation of dietary fibres (Figure 3). These SCFAs reach the bloodstream and can regulate gene transcription epigenetically, e.g. by blocking histone deacetylase activity (Stoler et al., 2023). In addition, SCFAs are reported to directly protect from ischaemic kidney damage in rodent models (Andrade‐Oliveira et al., 2015). Given that AKI‐susceptible proximal tubular cells exhibit limited glycolytic capacity, SCFAs may support lipid metabolism and thereby help meet their high energy demands (Scholz et al., 2021). In humans, high‐fibre diets promote bacterial production of SCFAs by altering the composition of the gut microbiome and have been shown to counteract obesity. Very strict high‐fibre diets supplemented with acarbose are reported to alleviate type 2 diabetes in patients (Koehler et al., 2024; Zhao et al., 2018). Besides high‐fibre diets, inulin‐derived prebiotics are reported to change the gut microbiome in favour of SCFA production in randomized controlled trials (Healey et al., 2018; Medawar et al., 2024).

Furthermore, gut bacteria account for the majority of H_2_S production, which can be enhanced by beneficial dietary interventions in rodent models (Lobel et al., 2020). In line with SAA restriction, microbially derived H_2_S may modify the host proteome post‐translationally, thereby augmenting protection from uraemic toxicity and disease progression in a rodent CKD model (Koehler et al., 2021; Lobel et al., 2020). As microbial sulfur metabolism is conserved in the human gut, these findings call for further research at the cross‐roads between microbiology, metabolism and molecular nephrology (Koehler et al., 2024; Wolf et al., 2022).

## 12 EMERGING PHARMACOLOGICAL METABOLIC THERAPIES FOR AKI: KETONE BODIES AND SGLT2 INHIBITORS

Although dietary interventions have consistently shown protective effects in preclinical models of AKI, their implementation in clinical practice remains challenging. Strict restrictive dietary regimens often present practical limitations such as low adherence, contraindications in frail or comorbid populations, and potential nutritional risks if not carefully supervised. To overcome these limitations, recent research has focused on endogenous metabolites capable of reproducing the benefits of dietary restriction in a more practical and controlled manner. Compounds such as spermidine, NAD^+^ boosters, and ketone bodies enhance renal resilience by promoting autophagy, reducing oxidative stress and inflammation, and preserving mitochondrial integrity – mimicking many of the adaptive responses triggered by fasting or CR but achievable through supplementation rather than actual prolonged dietary deprivation (Alhumaidi et al., 2025; Luo et al., 2023; Rojas‐Morales P et al., 2021).

Among these, ketone bodies have attracted particular interest in nephrology. These endogenous metabolites act as efficient energy substrates during metabolic stress and as signalling molecules that attenuate oxidative stress and inflammation, thereby protecting renal cells across diverse pathological contexts, including nephrotoxic, ischaemic and septic AKI (Kim et al., 2023; Rojas‐Morales P et al., 2021). Their benefits also extend to chronic kidney disease and hypertension, emphasizing their value in renal therapy (Rojas‐Morales P et al., 2021). Importantly, lifestyle interventions associated with health and longevity – such as fasting, CR and KDs – naturally enhance ketone body production, positioning them as a key mechanistic link between nutritional interventions and renal protection. Moreover, ketosis can be pharmacologically or nutritionally induced through exogenous ketone esters or salts, which are already available as dietary supplements, offering a feasible means to harness their therapeutic potential without the constraints of strict dietary restriction (Soto‐Mota et al., 2019).

Interestingly, similar metabolic and signalling effects can be achieved pharmacologically with sodium–glucose cotransporter 2 inhibitors (SGLT2i). Originally developed as glucose‐lowering agents, these drugs promote urinary glucose excretion, creating a mild systemic energy deficit that shifts metabolism toward enhanced fatty acid oxidation and ketogenesis. In parallel, SGLT2i activate nutrient‐sensing pathways such as AMPK and sirtuins, enhance mitochondrial efficiency, and reduce renal oxygen demand and inflammation – collectively recapitulating many renoprotective mechanisms of dietary restriction and ketone metabolism (Nakao et al., 2025; Upadhyay, 2024). Based on their protective effects in different organ systems, SGLT2i have also been classified as one of the most promising candidates among therapeutics tackling ageing‐associated disease in general (Kulkarni et al., 2022).

**FIGURE 4:**
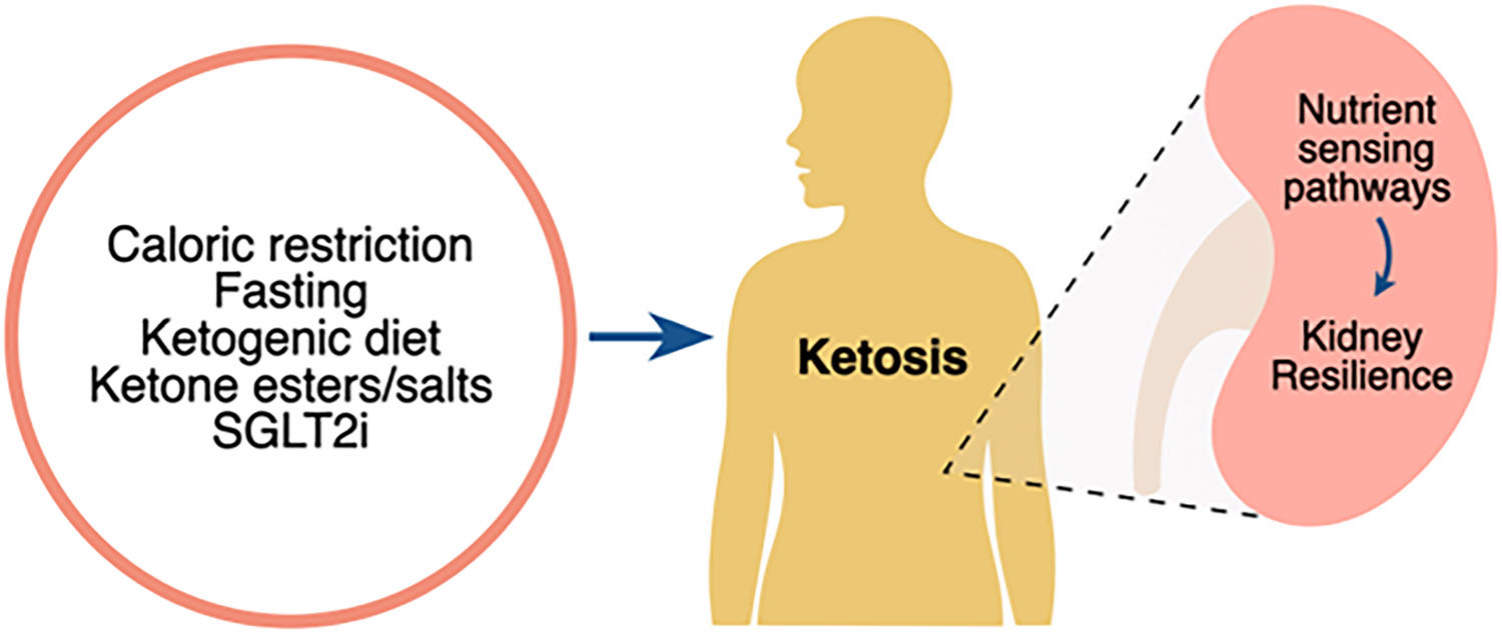
Ketosis as a metabolic therapy for AKI. Increasing endogenous ketone levels results in activation of nutrient sensing pathways and downstream cellular changes that ultimately defend the kidney from acute stress.

Given these shared pathways, the combined or alternative use of exogenous ketones and SGLT2i offers an appealing strategy to achieve the cellular and metabolic benefits of dietary interventions without their practical limitations. Both approaches modulate oxidative stress and inflammation, and promote cellular resilience. Thus, whether induced nutritionally or pharmacologically, therapeutic ketosis may provide a feasible, physiologically based approach to mitigate the burden of AKI (Figure 4).

## 13 TOWARD CLINICAL IMPLEMENTATION: CHALLENGES AND HUMAN EVIDENCE

Despite decades of (pre‐)clinical drug development, therapeutic approaches for AKI in the clinic remain supportive and pharmacotherapies are still lagging behind (Ostermann et al., 2025). In contrast, dietary interventions may be used as so‐called pre‐rehabilitation strategies capable of achieving clinically favourable outcomes with minimal time investment by patients, particularly when formula diets are employed (Koehler et al., 2024). As novel non‐pharmacological approaches, such as dietary interventions, show an immense potential in preserving kidney function in various animal models of AKI, they may add substantially to our therapeutic toolkit. In this context, cardiac surgery and kidney transplantation may serve as human models of renal IRI.

Despite the immense potential of dietary preconditioning observed in rodent models, the primary endpoint – the median serum creatinine increase after 24 h – did not differ between preoperative dietary preconditioned patients adhering to CR compared to non‐preconditioned control patients examined in a randomized controlled clinical trial among patients undergoing cardiac surgery (Grundmann et al., 2018). However, CR efficiently prevented a rise in median creatinine at 48 h. A subgroup analysis revealed most profound beneficial effects of CR‐preconditioning in male patients and patients with a BMI >25 kg/m^2^ (Grundmann et al., 2018). A second study using CR to prevent AKI in patients before cardiac catheter examinations, again revealed mild protective effects in subgroup analyses (Grundmann et al., 2020). While caloric preconditioning has some protective effects, providing full nutrition after the onset of an acute critical illness may increase morbidity and mortality in critically ill patients (de Man et al., 2024). This finding is in line with several clinical trials reporting that early nutrition in the ICU is associated with higher risk of both AKI and RRT (de Man et al., 2024; Druml et al., 2024). Conversely, ketosis, for example, by ketogenic diets, ketone bodies or limited caloric intake, has shown beneficial effects in clinical trials involving critically ill ICU patients (McNelly et al., 2023; Rahmel et al., 2024).

When restricting dietary intake of methionine and cysteine by 80%, no beneficial effects on the rate of AKI were reported in a single‐centre, randomized controlled clinical trial (Osterholt et al., 2022). There are various reasons that may account for this observed discrepancy between rodents and humans. First, enrolled patients in this trial were at a relatively low risk for RRT, which was mirrored by a low Cleveland Clinic Foundation score, and, in turn, only rarely developed advanced kidney failure (Osterholt et al., 2022; Thakar et al., 2005). In contrast, the rodent model uses unilateral nephrectomy combined with a prolonged contralateral ischaemia resulting in a robust and pronounced renal failure (Koehler et al., 2022). Second, the used collagen‐based protein source not only reduced SAA content, but additionally led to quantitative alterations in other amino acids as well. Hence, the applied formula diet cannot be considered equivalent to the diets used in animal studies. Third and most importantly, the rodent studies indicate that a more profound reduction in the uptake of SAA, ideally a full depletion of methionine and cysteine, is needed and is superior in protecting kidney function (Koehler et al., 2022).

**FIGURE 5:**
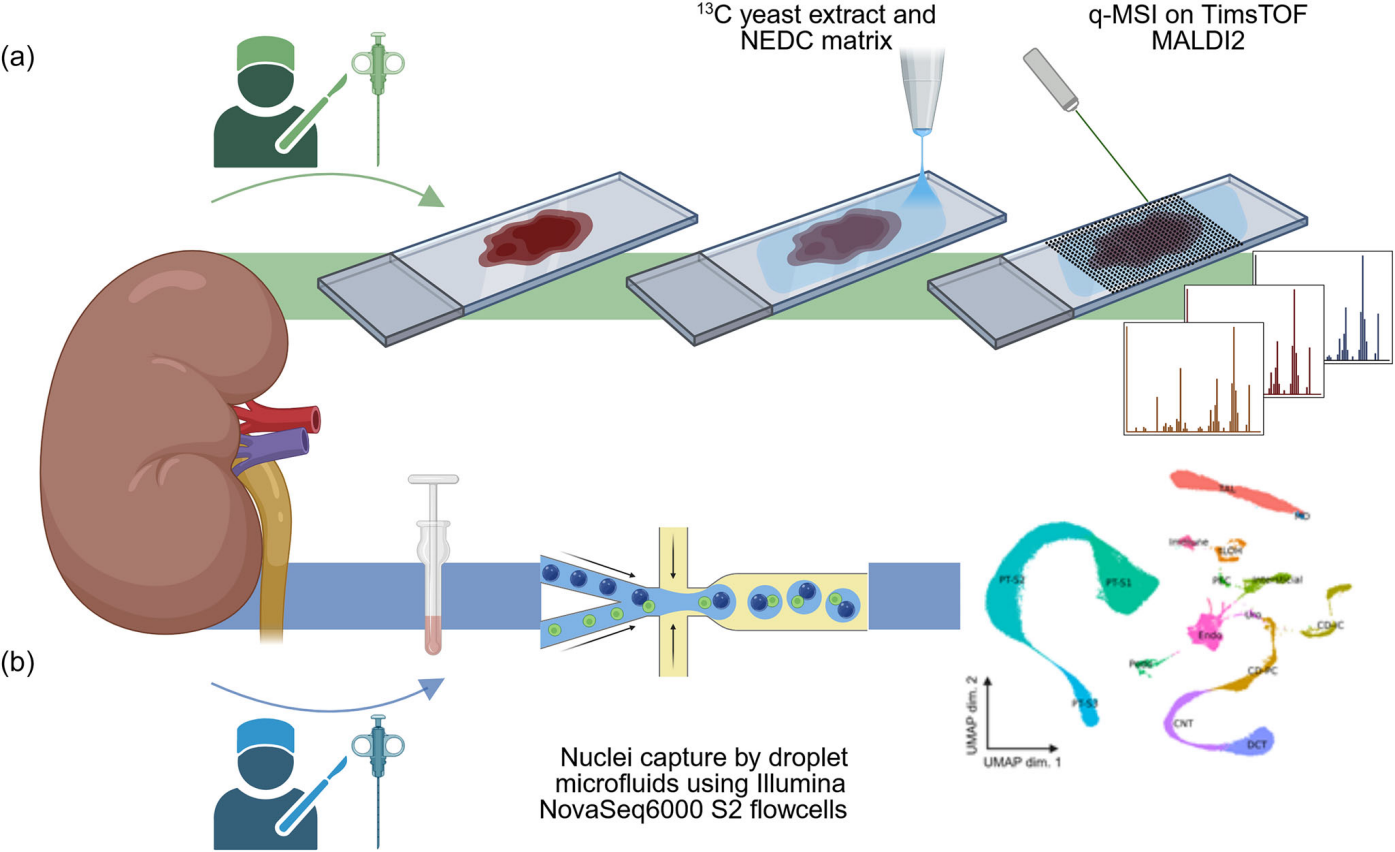
Kidney biopsy samples of dietary preconditioned kidney donors are collected per protocol of the DILKID‐Trial during the transplantation procedure. Kidney biopsy samples are used for (a) quantitative matrix‐assisted laser desorption/ionization mass spectrometry imaging and (b) single nucleus RNA sequencing to recapitulate the cell type specific diet‐induced metabolic and genetic response. NEDC: *N*‐(1‐naphthyl) ethylenediamine dihydrochloride. Figure created with BioRender.com.

Thus, the study of the underlying molecular mechanisms that orchestrate diet‐induced kidney protection in human material is crucial at this point and will help to pave the way for a successful transfer in the clinic, but also to identify druggable targets. Of note, the beneficial dietary interventions share hallmarks of cellular stress‐resistance in preclinical models, which is a promising starting point to delve into the study of molecular mechanisms in human material (Figure 3). Triggered by this idea, we initiated two randomized controlled pilot trials employing the discussed dietary interventions during living kidney donation, which not only facilitated feasibility and safety analyses, but more importantly allowed the collection of human kidney samples during transplant surgery. This is an important pre‐requisite to recapitulate molecular mechanisms of kidney protection in human material (Koehler et al., 2024). The CR_LSP‐Trial (NCT02745444) examines a low‐calorie formula diet, whereas the DILKID‐Trial (NCT05709600) is a four‐arm trial using FMD, a low‐SAA diet and KD compared to a control diet. As the susceptibility of the different renal cell types regarding AKI varies strongly, we can now add novel powerful techniques such as single nucleus RNA sequencing and quantitative matrix‐assisted laser desorption/ionization mass spectrometry imaging to facilitate cell‐type‐specific analysis to overcome AKI's heterogenic pathophysiology (Figure 5) (Koehler et al., 2024; Scholz et al., 2021; Wang et al., 2025). In addition to the identification of novel pharmacological approaches, these translational pilot trials may help to close the gap from bench to bedside in nephrology for the beneficial dietary interventions highlighted in this review (Koehler et al., 2024). Cell‐type‐specific cysteine metabolism may be a promising starting‐point both for elucidating the mechanisms underlying diet‐induced kidney protection and for developing novel therapeutic strategies (Koehler et al., 2022, 2024).

## AUTHOR CONTRIBUTIONS

No generative artificial intelligence (GAI) tools were used in the preparation of this manuscript. Felix C. Koehler wrote the manuscript. Pedro Rojas‐Morales and Roman‐UlrichMüller revised the manuscript. All authors have read and approved the final version of this manuscript and agree to be accountable for all aspects of the work in ensuring that questions related to the accuracy or integrity of any part of the work are appropriately investigated and resolved. All persons designated as authors qualify for authorship, and all those who qualify for authorship are listed.

## CONFLICT OF INTEREST

F.C.K reports consulting fees from Atriva Therapeutics GmbH, outside the submitted work.

## Supporting information



Supplementary Table S1.
