# Supplementary material for: Dietary interventions in acute kidney injury: From molecular mechanism to clinical trials
Source: Exp Physiol. 2026 Mar 25:10.1113/EP092817. Online ahead of print. doi: 10.1113/EP092817 (PMC13394128; doi:10.1113/EP092817)
Supplement: Supplementary file 1 — Supplementary Table S1. [file EPH-9999-0-s001.pdf]

Hallmark of cellular stress-resistance	Dietary regimen	Potential effect	
		Preclinical models	Humans
Genomic stability	Caloric restriction	(1-3)	(4)
	Periodic Fasting	(5)	
	Ketogenic diet	(6)	(6)
	Low-SAA diet	(7-9)	
Epigenetic stability	Caloric restriction	(10, 11)	(12)
	Periodic Fasting	(5, 13, 14)	
	Ketogenic diet		(15)
	Low-SAA diet	(16, 17)	
Proteostasis	Caloric restriction	(18, 19)	(19)
	Periodic Fasting	(18)	(20)
	Ketogenic diet	(21)	
	Low-SAA diet	(18, 22-24)	
Nutrient sensing	Caloric restriction		(25-27)
	Periodic Fasting	(5, 28-30)	(31)
	Ketogenic diet	(32-34)	(35)
	Low-SAA diet	(23, 36-39)	(40)
Mitochondrial function	Caloric restriction	(41, 42)	(41)
	Periodic Fasting	(18, 43)	
	Ketogenic diet	(44)	(45)
	Low-SAA diet	(18, 42)	
Gut microbiota remodelling	Caloric restriction	(46-51)	(52, 53)
	Periodic Fasting	(54, 55)	
	Ketogenic diet	(50, 51, 56)	(57, 58)
	Low-SAA diet	(59)	

Supplementary Table 1: Summary of reference literature describing diet-induced effects on hallmarks of cellular stress-resistance observed in rodent models in addition to human adhering to specific dietary pattern in clinical trials (Figure 3).

Supplementary References:

1. Cabelof DC, Yanamadala S, Raffoul JJ, Guo Z, Soofi A, and Heydari AR. Caloric restriction promotes genomic stability by induction of base excision repair and reversal of its age-related decline. DNA Repair (Amst) 2003; 2: 295-307
2. Kisby GE, Kohama SG, Olivas A, et al. Effect of caloric restriction on base-excision repair (BER) in the aging rat brain. Exp Gerontol 2010; 45: 208-216
3. Sanz A, Caro P, Ibañez J, Gómez J, Gredilla R, and Barja G. Dietary restriction at old age lowers mitochondrial oxygen radical production and leak at complex I and oxidative DNA damage in rat brain. J Bioenerg Biomembr 2005; 37: 83-90
4. Heilbronn LK, de Jonge L, Frisard MI, et al. Effect of 6-month calorie restriction on biomarkers of longevity, metabolic adaptation, and oxidative stress in overweight individuals: a randomized controlled trial. Jama 2006; 295: 1539-1548
5. Longo VD, Di Tano M, Mattson MP, and Guidi N. Intermittent and periodic fasting, longevity and disease. Nat Aging 2021; 1: 47-59
6. Wei SJ, Schell JR, Chocron ES, et al. Ketogenic diet induces p53-dependent cellular senescence in multiple organs. Sci Adv 2024; 10: eado1463
7. Zhang Y, Jelleschitz J, Grune T, et al. Methionine restriction - Association with redox homeostasis and implications on aging and diseases. Redox Biol 2022; 57: 102464
8. Tanrikulu-Kucuk S, and Ademoglu E. Dietary restriction of amino acids other than methionine prevents oxidative damage during aging: involvement of telomerase activity and telomere length. Life Sci 2012; 90: 924-928
9. Bárcena C, Quirós PM, Durand S, et al. Methionine Restriction Extends Lifespan in Progeroid Mice and Alters Lipid and Bile Acid Metabolism. Cell Rep 2018; 24: 2392-2403
10. Grande de França NA, Rolland Y, Guyonnet S, and de Souto Barreto P. The role of dietary strategies in the modulation of hallmarks of aging. Ageing Res Rev 2023; 87: 101908
11. Gong H, Qian H, Ertl R, et al. Histone modifications change with age, dietary restriction and rapamycin treatment in mouse brain. Oncotarget 2015; 6: 15882-15890
12. Waziry R, Ryan CP, Corcoran DL, et al. Effect of long-term caloric restriction on DNA methylation measures of biological aging in healthy adults from the CALERIE trial. Nat Aging 2023; 3: 248-257
13. Byun S, Seok S, Kim YC, et al. Fasting-induced FGF21 signaling activates hepatic autophagy and lipid degradation via JMJD3 histone demethylase. Nat Commun 2020; 11: 807
14. Seok S, Kim YC, Byun S, et al. Fasting-induced JMJD3 histone demethylase epigenetically activates mitochondrial fatty acid β-oxidation. J Clin Invest 2018; 128: 3144-3159

15. Izquierdo AG, Lorenzo PM, Costa-Fraga N, et al. Epigenetic Aging Acceleration in Obesity Is Slowed Down by Nutritional Ketosis Following Very Low-Calorie Ketogenic Diet (VLCKD): A New Perspective to Reverse Biological Age. Nutrients 2025; 17:
16. Hernández-Arciga U, Stamenkovic C, Yadav S, et al. Dietary methionine restriction started late in life promotes healthy aging in a sex-specific manner. Sci Adv 2025; 11: eads1532
17. Mentch SJ, Mehrmohamadi M, Huang L, et al. Histone Methylation Dynamics and Gene Regulation Occur through the Sensing of One-Carbon Metabolism. Cell Metab 2015; 22: 861-873
18. Koehler FC, Fu CY, Späth MR, et al. A systematic analysis of diet-induced nephroprotection reveals overlapping changes in cysteine catabolism. Transl Res 2022:
19. Tiku V, Jain C, Raz Y, et al. Small nucleoli are a cellular hallmark of longevity. Nat Commun 2017; 8: 16083
20. Caffa I, Spagnolo V, Vernieri C, et al. Fasting-mimicking diet and hormone therapy induce breast cancer regression. Nature 2020; 583: 620-624
21. Liśkiewicz D, Liśkiewicz A, Grabowski M, et al. Upregulation of hepatic autophagy under nutritional ketosis. J Nutr Biochem 2021; 93: 108620
22. Jonsson WO, Margolies NS, and Anthony TG. Dietary Sulfur Amino Acid Restriction and the Integrated Stress Response: Mechanistic Insights. Nutrients 2019; 11:
23. Stone KP, Ghosh S, Kovalik JP, et al. The acute transcriptional responses to dietary methionine restriction are triggered by inhibition of ternary complex formation and linked to Erk1/2, mTOR, and ATF4. Sci Rep 2021; 11: 3765
24. Zou K, Rouskin S, Dervishi K, et al. Life span extension by glucose restriction is abrogated by methionine supplementation: Cross-talk between glucose and methionine and implication of methionine as a key regulator of life span. Sci Adv 2020; 6: eaba1306
25. Fontana L, Villareal DT, Das SK, et al. Effects of 2-year calorie restriction on circulating levels of IGF-1, IGF-binding proteins and cortisol in nonobese men and women: a randomized clinical trial. Aging Cell 2016; 15: 22-27
26. Opstad TB, Sundfør T, Tonstad S, and Seljeflot I. Effect of intermittent and continuous caloric restriction on Sirtuin1 concentration depends on sex and body mass index. Nutr Metab Cardiovasc Dis 2021; 31: 1871-1878
27. Roggerio A, Strunz CMC, Pacanaro AP, et al. Gene Expression of Sirtuin-1 and Endogenous Secretory Receptor for Advanced Glycation End Products in Healthy and Slightly Overweight Subjects after Caloric Restriction and Resveratrol Administration. Nutrients 2018; 10:

28. Brandhorst S, Choi IY, Wei M, et al. A Periodic Diet that Mimics Fasting Promotes Multi-System Regeneration, Enhanced Cognitive Performance, and Healthspan. Cell Metab 2015; 22: 86-99
29. Yuliyanasari N, Rejeki PS, Hidayati HB, Subsomwong P, and Miftahussurur M. The effect of intermittent fasting on preventing obesity-related early aging from a molecular and cellular perspective. J Med Life 2024; 17: 261-272
30. Fry JL, Al Sayah L, Weisbrod RM, et al. Vascular Smooth Muscle Sirtuin-1 Protects Against Diet-Induced Aortic Stiffness. Hypertension 2016; 68: 775-784
31. Wei M, Brandhorst S, Shelehchi M, et al. Fasting-mimicking diet and markers/risk factors for aging, diabetes, cancer, and cardiovascular disease. Sci Transl Med 2017; 9:
32. Mavropoulos JC, Buschemeyer WC, 3rd, Tewari AK, et al. The effects of varying dietary carbohydrate and fat content on survival in a murine LNCaP prostate cancer xenograft model. Cancer Prev Res (Phila) 2009; 2: 557-565
33. Dabke P, and Das AM. Mechanism of Action of Ketogenic Diet Treatment: Impact of Decanoic Acid and Beta-Hydroxybutyrate on Sirtuins and Energy Metabolism in Hippocampal Murine Neurons. Nutrients 2020; 12:
34. Fan SZ, Lin CS, Wei YW, et al. Dietary citrate supplementation enhances longevity, metabolic health, and memory performance through promoting ketogenesis. Aging Cell 2021; 20: e13510
35. Rajakumar G, Cagigas ML, Wang T, et al. Effect of ketogenic diets on insulin-like growth factor (IGF)-1 in humans: A systematic review and meta-analysis. Ageing Res Rev 2024; 102: 102531
36. Kitada M, Xu J, Ogura Y, Monno I, and Koya D. Mechanism of Activation of Mechanistic Target of Rapamycin Complex 1 by Methionine. Front Cell Dev Biol 2020; 8: 715
37. Plummer JD, and Johnson JE. Intermittent methionine restriction reduces IGF-1 levels and produces similar healthspan benefits to continuous methionine restriction. Aging Cell 2022; 21: e13629
38. Malloy VL, Krajcik RA, Bailey SJ, Hristopoulos G, Plummer JD, and Orentreich N. Methionine restriction decreases visceral fat mass and preserves insulin action in aging male Fischer 344 rats independent of energy restriction. Aging Cell 2006; 5: 305-314
39. Takenaka A, Oki N, Takahashi SI, and Noguchi T. Dietary restriction of single essential amino acids reduces plasma insulin-like growth factor-I (IGF-I) but does not affect plasma IGF-binding protein-1 in rats. J Nutr 2000; 130: 2910-2914
40. Richie JP, Jr., Sinha R, Dong Z, et al. Dietary Methionine and Total Sulfur Amino Acid Restriction in Healthy Adults. J Nutr Health Aging 2023; 27: 111-123

41. Späth MR, Hoyer-Allo KJR, Seufert L, et al. Organ Protection by Caloric Restriction Depends on Activation of the De Novo NAD+ Synthesis Pathway. J Am Soc Nephrol 2023; 34: 772-792
42. Hine C, Harputlugil E, Zhang Y, et al. Endogenous hydrogen sulfide production is essential for dietary restriction benefits. Cell 2015; 160: 132-144
43. Levine DC, Kuo HY, Hong HK, et al. NADH inhibition of SIRT1 links energy state to transcription during time-restricted feeding. Nat Metab 2021; 3: 1621-1632
44. Koronowski KB, Greco CM, Huang H, et al. Ketogenesis impact on liver metabolism revealed by proteomics of lysine β-hydroxybutyrylation. Cell Rep 2021; 36: 109487
45. Luukkonen PK, Dufour S, Lyu K, et al. Effect of a ketogenic diet on hepatic steatosis and hepatic mitochondrial metabolism in nonalcoholic fatty liver disease. Proc Natl Acad Sci U S A 2020; 117: 7347-7354
46. Lee J, d'Aigle J, Atadja L, et al. Gut Microbiota-Derived Short-Chain Fatty Acids Promote Poststroke Recovery in Aged Mice. Circ Res 2020; 127: 453-465
47. Kurup K, Matyi S, Giles CB, et al. Calorie restriction prevents age-related changes in the intestinal microbiota. Aging (Albany NY) 2021; 13: 6298-6329
48. Gharaie S, Noel S, and Rabb H. Gut Microbiome and AKI: Roles of the Immune System and Short-Chain Fatty Acids. Nephron 2020; 144: 662-664
49. Gharaie S, Lee K, Newman-Rivera AM, et al. Microbiome modulation after severe acute kidney injury accelerates functional recovery and decreases kidney fibrosis. Kidney Int 2023; 104: 470-491
50. Rojas-Morales P, León-Contreras JC, Sánchez-Tapia M, et al. A ketogenic diet attenuates acute and chronic ischemic kidney injury and reduces markers of oxidative stress and inflammation. Life Sci 2022; 289: 120227
51. Fabbiano S, Suárez-Zamorano N, Chevalier C, et al. Functional Gut Microbiota Remodeling Contributes to the Caloric Restriction-Induced Metabolic Improvements. Cell Metab 2018; 28: 907-921.e907
52. Mohr AE, Sweazea KL, Bowes DA, et al. Gut microbiome remodeling and metabolomic profile improves in response to protein pacing with intermittent fasting versus continuous caloric restriction. Nat Commun 2024; 15: 4155
53. Li L, Li R, Tian Q, et al. Effects of healthy low-carbohydrate diet and time-restricted eating on weight and gut microbiome in adults with overweight or obesity: Feeding RCT. Cell Rep Med 2024; 5: 101801
54. Rangan P, Choi I, Wei M, et al. Fasting-Mimicking Diet Modulates Microbiota and Promotes Intestinal Regeneration to Reduce Inflammatory Bowel Disease Pathology. Cell Rep 2019; 26: 2704-2719.e2706

55. Luo M, Wang Q, Sun Y, et al. Fasting-mimicking diet remodels gut microbiota and suppresses colorectal cancer progression. NPJ Biofilms Microbiomes 2024; 10: 53

56. Ang QY, Alexander M, Newman JC, et al. Ketogenic Diets Alter the Gut Microbiome Resulting in Decreased Intestinal Th17 Cells. Cell 2020; 181: 1263-1275.e1216

57. Güzey Akansel M, Baş M, Gençalp C, et al. Effects of the Ketogenic Diet on Microbiota Composition and Short-Chain Fatty Acids in Women with Overweight/Obesity. Nutrients 2024; 16:

58. Rahmel T, Effinger D, Bracht T, et al. An open-label, randomized controlled trial to assess a ketogenic diet in critically ill patients with sepsis. Sci Transl Med 2024; 16: eadn9285

59. Koehler FC, Späth MR, Meyer AM, and Müller RU. Fueling the success of transplantation through nutrition: recent insights into nutritional interventions, their interplay with gut microbiota and cellular mechanisms. Curr Opin Organ Transplant 2024:
